# Interaction of major monoterpenes from *Rosmarinus officinalis* L. essential oil with the human serotonin transporter: molecular docking, radioligand binding, and functional uptake studies

**DOI:** 10.3389/fphar.2026.1923534

**Published:** 2026-09-25

**Authors:** Carolina Valle, Kerim Segura, Pilar Cárdenas, Nelson Osses, Jaime Mella, Patricio Iturriaga-Vásquez, José A. Pino, Michel Lapier

**Affiliations:** 1 Escuela de Química y Farmacia, Facultad de Farmacia, Universidad de Valparaíso, Valparaíso, Chile; 2 Laboratorio de Bioquímica y Farmacología Molecular, Departamento de Ciencias Biomédicas, Facultad de Ciencias de La Vida, Universidad Viña del Mar, Viña del Mar, Chile; 3 Laboratorio de Farmacología Molecular y Química Medicinal, Departamento de Ciencias Químicas y Recursos Naturales, Facultad de Ingeniería y Ciencias, Universidad de La Frontera, Temuco, Chile; 4 Instituto de Química, Facultad de Ciencias, Pontificia Universidad Católica de Valparaíso, Valparaíso, Chile; 5 Center for Interdisciplinary Research in Biomedicine, Biotechnology and Wellbeing (CID3B), Pontificia Universidad Católica de Valparaíso, Valparaíso, Chile; 6 Instituto de Química, Facultad de Ciencias, Universidad de Valparaíso, Valparaíso, Chile; 7 Center for Research, Development and Innovation of Bioactive Products (CinBio), School of Chemistry and Pharmacy, Faculty of Pharmacy, University of Valparaíso, Valparaíso, Chile

**Keywords:** 1,8-cineole, camphor, molecular docking, rosemary essential oil, serotonin transporter, SERT function, α-pinene

## Abstract

Major depressive disorder remains a leading cause of disability worldwide, and current antidepressants are limited by delayed therapeutic onset, incomplete efficacy, and adverse effects. Although *Rosmarinus officinalis* L. essential oil and its major monoterpenes exhibit antidepressant-like and anxiolytic properties, their molecular mechanisms remain unclear. This study investigated whether the principal rosemary monoterpenes, α-pinene, camphor, and 1,8-cineole, directly interact with the human serotonin transporter (hSERT) and its functional effects. Molecular docking simulations targeting the orthosteric (S1) and allosteric (S2) binding sites were combined with [^3^H]-paroxetine binding displacement assays and SERT-mediated uptake experiments based on the fluorescent serotonin analog FFN246 in HEK293 cells expressing YFP-tagged hSERT. Docking analyses predicted plausible binding poses for all three monoterpenes within both the S1 and S2 regions, although with less favorable docking scores than fluoxetine. Radioligand displacement assays provided experimental evidence that all three compounds interact with hSERT, producing concentration-dependent displacement of [^3^H]-paroxetine, with α-pinene displaying the highest potency, followed by 1,8-cineole and camphor. Functional assays further showed that all monoterpenes produced significant but incomplete inhibition of transporter-mediated FFN246 uptake, even at high micromolar concentrations, indicating partial rather than complete transporter blockade. Together, these findings identify hSERT as a previously unrecognized molecular target of rosemary monoterpenes, suggesting that these compounds may act as partial inhibitors of serotonin transport. These results provide new molecular insights into the potential mechanism that may contribute to the reported neurobehavioral effects of rosemary, and support further investigation of these compounds in serotonergic signaling and neurotransmission.

## 1 Introduction

Major depressive disorder (MDD) is one of the leading causes of disability worldwide and constitutes a major public health challenge due to its high prevalence, chronicity, and substantial socioeconomic burden (Mental Disorders Collaborators, 2022 Mental Disorders Mental Disorders Collaborators, 2022; Malhi and Mann, 2018). Although several pharmacological treatments are currently available, including selective serotonin reuptake inhibitors (SSRIs), therapeutic efficacy remains suboptimal, with a significant proportion of patients failing to achieve remission or experiencing adverse effects that compromise treatment adherence (Cipriani et al., 2018; Clayton et al., 2016; Cowen, 2024). Consequently, the identification of novel pharmacological strategies, including natural bioactive molecules, capable of modulating serotonergic neurotransmission through alternative mechanisms, has emerged as a priority in neuropsychopharmacology (Fonseca et al., 2023; Krystal et al., 2019; Machado et al., 2009; Niello et al., 2020; Weston-Green et al., 2021).

The serotonin transporter (SERT) is the principal regulator of extracellular serotonin levels in the central nervous system and represents the primary molecular target of SSRIs (Torres et al., 2003; Kristensen et al., 2011; Coleman et al., 2016). SERT belongs to the solute carrier 6 (SLC6A4) family of neurotransmitter transporters and mediates the reuptake of serotonin from the synaptic cleft into presynaptic neurons, thereby terminating serotonergic signaling and maintaining neurotransmitter homeostasis (Kristensen et al., 2011; Torres et al., 2003). Structural and pharmacological studies have identified that SERT contains a central orthosteric binding site (S1), responsible for substrate recognition and transport, as well as an extracellular allosteric site (S2) capable of modulating transporter activity and ligand dissociation kinetics (Zhong et al., 2012; Coleman et al., 2016; Plenge et al., 2021; Salomon et al., 2023). In addition, the classical pharmacological inhibition produced by most SSRIs is primarily explained by competitive binding at S1, whereas the clinically relevant allosteric behavior is most clearly established for escitalopram at S2 (Coleman et al., 2016; Coleman and Gouaux, 2018; Plenge et al., 2021).

Despite the clinical success of SSRIs, their therapeutic use is associated with several limitations, including delayed onset of action, incomplete efficacy, and undesirable side effects (Cipriani et al., 2018; Clayton et al., 2016; Cowen, 2024; Masand and Gupta, 1999). These limitations have stimulated interest in naturally occurring compounds with neuroactive properties, particularly those derived from medicinal and aromatic plants (Fonseca et al., 2023; Rahbardar and Hosseinzadeh, 2020). Among these, Rosemary (*Rosmarinus officinalis* L.) has attracted considerable attention because of its broad spectrum of neuroprotective, anxiolytic, antidepressant-like, and cognitive-enhancing effects observed in both preclinical and clinical studies (Araki et al., 2020; Habtemariam, 2016; Moss et al., 2018; Rahbardar and Hosseinzadeh, 2020). Rosemary essential oil is a complex phytochemical mixture dominated by monoterpenes, including α-pinene, 1,8-cineole, and camphor (Jalali-Heravi et al., 2011; Jiang et al., 2011; Micić et al., 2021), which have individually demonstrated neuroactive properties in experimental models (Ceremuga et al., 2017; Guzmán-Gutiérrez et al., 2015; Park et al., 2021; Salama et al., 2021). Behavioral studies have shown that α-pinene and 1,8-cineole can produce antidepressant-like and anxiolytic-like effects in animal models, whereas rosemary extracts and essential oils have been reported to influence monoaminergic neurotransmission (Ceremuga et al., 2017; Guzmán-Gutiérrez et al., 2015; Machado et al., 2009; 2013; Niu et al., 2025). However, the molecular mechanisms underlying these neuropsychopharmacological actions remain largely unknown (Fonseca et al., 2023; Rahbardar and Hosseinzadeh, 2020).

Recent reviews and experimental studies suggest that essential oils and their constituent terpenoids may interact with multiple central nervous system targets, including GABAergic, dopaminergic, glutamatergic, endocannabinoid, and monoaminergic pathways (Fonseca et al., 2023; López et al., 2017; Rahbardar and Hosseinzadeh, 2020). Nevertheless, most investigations on rosemary and its major constituents have focused primarily on behavioral outcomes, neurochemical alterations, or indirect pharmacological effects (Araki et al., 2020; Machado et al., 2009; 2013; Niu et al., 2025; Sayorwan, 2013). Despite the established role of SERT regulation in antidepressant action (Masand and Gupta, 1999), to date, no study has comprehensively evaluated whether the principal monoterpenes present in rosemary essential oil directly interact with SERT, nor has the potential contribution of orthosteric and allosteric SERT binding to their pharmacological actions been systematically investigated. This lack of mechanistic information represents an important gap in the current understanding of rosemary neuropharmacology (Fonseca et al., 2023; Rahbardar and Hosseinzadeh, 2020). Addressing these questions is essential to achieve a more comprehensive role of these natural compounds in antidepressant activities.

Therefore, the present study aimed to investigate whether the major monoterpenes of *R. officinalis* L. essential oil, namely, α-pinene, camphor, and 1,8-cineole, interact physically and functionally with the human serotonin transporter. To address this objective, we combined molecular docking simulations to explore potential ligand-binding poses within the orthosteric (S1) and extracellular (S2) regions of hSERT with radioligand displacement assays and hSERT functional experiments. Our results show that all three monoterpenes produce concentration-dependent displacement of [^3^H]-paroxetine binding, with IC_50_ values in the low micromolar range, and significantly reduce hSERT-mediated FFN246 uptake, while molecular docking predicts plausible interactions within both the S1 and S2 regions of the transporter. Collectively, these findings provide evidence that the major monoterpenes of *R. officinalis* L. interact physically and functionally with hSERT and provide a mechanistic framework linking the neurobehavioral effects of *R. officinalis* L. to direct modulation of serotonergic neurotransmission.

## 2 Materials and methods

### 2.1 Molecular docking

The crystal structures of the human serotonin transporter (hSERT) were obtained from the Protein Data Bank (PDB IDs: 5I71 and 5I73), containing citalopram bound to the orthosteric (S1) and allosteric (S2) sites, respectively (Coleman et al., 2016). Protein structures were prepared in Schrödinger Maestro 14.3 (Schrödinger LLC) using the Protein Preparation Wizard by removing water molecules, adding hydrogen atoms, assigning bond orders, adjusting protonation states at physiological pH, and minimizing the structures with the OPLS4 force field.

Camphor, 1,8-cineole, α-pinene, fluoxetine, and citalopram were prepared with LigPrep (Schrödinger LLC) from PubChem SMILES structures. Protonation states were generated using Epik Classic (pH 7.0 ± 2.0), followed by geometry optimization with the OPLS4 force field.

Docking grids were centered on the co-crystallized citalopram molecules to define the orthosteric (S1) and allosteric (S2) binding sites. Molecular docking was performed using the Glide Extra Precision (XP) protocol (Schrödinger LLC). Binding poses were ranked according to the GlideScore scoring function, and the top-ranked pose for each ligand was selected for interaction analysis. Hydrogen bonds, hydrophobic contacts, aromatic interactions, docking scores, and interacting residues were analyzed using Maestro.

The docking protocol was validated by redocking the co-crystallized citalopram molecules. The predicted poses closely reproduced the crystallographic conformations, yielding RMSD values of 0.91 Å for the S1 site (5I71) and 0.67 Å for the S2 site (5I73), both below the accepted validation threshold of 2.0 Å (Supplementary Figure S1).

### 2.2 Cell culture and transfections

Human Embryonic Kidney 293 cells (HEK) were purchased from American Type Culture Collection. HEK cells were cultured in DMEM medium supplemented with 10% fetal bovine serum (FBS, Sigma-Aldrich), 1 mM L-glutamine, 100 U/mL penicillin and 100 μg/mL streptomycin (Sigma-Aldrich). The human SERT cDNA with the N-terminal fused to YFP was obtained from Harald Sitte laboratory (Addgene plasmid, Cat. N° 70,103) (Sucic et al., 2010), and used to transfect HEK cells with Lipofectamine 2000 (Invitrogen). Transient transfections were performed for 24 h in 6-well tissue culture plates, and cells overexpressing YFP-SERT (HEK-SERT-YFP cells) were verified using epifluorescence microscopy, and then divided to 6-well, 24-well or 96-well tissue culture plates and maintained in supplemented DMEM media for 24–48 h before binding assays and functional experiments.

### 2.3 Binding protocol of [^3^H]-Paroxetine on HEK-SERT cells

HEK-SERT-YFP cells were diluted in a storage buffer solution of Tris-HCl 50 mM (pH 7.4), EDTA 0.5 mM, MgCl2 10 mM and 10% sucrose, which was finally stored at −80 °C. Each Eppendorf tube was incubated with 50 mM Tris HCl buffer (pH 7.4), 120 mM NaCl, 5 mM KCl and the chemical compounds under study: camphor (USP, Cat. N^o^. 10877505), 1,8-cineole (Cat. N^o^. C80601), and α-pinene (Cat. N^o^. 268070) (Sigma-Aldrich), using increasing concentrations in presence of 2 nM of [^3^H]-paroxetine (specific activity 23.1 Ci/mmol, Code NET86925UC, PerkinElmer) in a final volume of 250 µL. Non-specific binding was determined using 25 mM fluoxetine (Sigma-Aldrich). After 30 min at 27 °C, the incubation was stopped by rapid filtration on a Whatman GF/C filter preabsorbed in 0.5% polyethylenimine (PEI), washed with cold working buffer solution (3 × 3 mL), filtered, and scintillation liquid was added. The radioactivity was measured by liquid scintillation spectrometry (MicroBeta 2450 microplate counter, PerkinElmer). Each graph obtained in the experiments was carried out in triplicate.

### 2.4 Evaluation of SERT activity via fluorometric assay

Recently, the false fluorescent neurotransmitter FFN246 has been used as a selective fluorescent substrate of SERT in heterologous systems and acute brain slices (Henke et al., 2018). Thus, FFN246 uptake can be used to test SERT function. Briefly, HEK cells overexpressing hSERT-YFP were seeded in 24-well plates and allowed to proliferate in growth medium for ∼2 days at 37 °C to reach confluence. At the start of the experiment, the growth medium was aspirated, wells were washed with 400 μL of PBS, and then treated for 10 min at 37 °C with: (1) 400 μL of uptake buffer (in mM: 4 Tris base, 6.25 HEPES, 120 NaCl, 5 KCl, 1.25 CaCl_2_, 1.25 MgSO_4_, 0.6 ascorbic acid, and 5.6 glucose, pH 7.4) with DMSO (control/vehicle, 0.2% v/v); (2) 400 μL of increasing concentration of 1,8-cineole, α-pinene, and camphor (Sigma-Aldrich) prepared in uptake buffer (10–600 μM); and (3) 400 μL of SERT inhibitor (0.5 μM fluoxetine). SERT functional experiments were initiated after probe addition (FFN246, 5 μM; Glixx Laboratories) and incubation for 20 min at 37 °C. The experiments were terminated by three rapid PBS washes. Fluorescent signals of intracellular FFN246 and SERT-YFP were recorded by epifluorescence microscopy (Nikon Diaphot), using a UV-2A cube (ex 330–380 nm/em 420 nm cut-on) for FFN246 and a B-2A cube (ex 450–490 nm/em 515 nm cut-on) for SERT-YFP.

### 2.5 Data analysis

Fluorescence signals (FFN246 and SERT-YFP) were analysed through the integrated density (IntDen), and corrected to background using at least 25 regions of interest (ROIs) with ImageJ software v1.54 (NIH) (Schneider et al., 2012). In addition, intracellular FFN246 IntDen (FFN246 uptake) was normalized to SERT-YFP IntDen, indicating expression levels of the transporter (FFN246 uptake/YFP). FFN246 uptake/YFP data are presented as mean ± SD obtained in at least 3 independent biological experiments performed on different days. The vehicle control, fluoxetine, and camphor (10–200 μM) groups were evaluated in five independent experiments (n = 5); camphor (400 μM) and all concentrations of 1,8-cineole and α-pinene were evaluated in four independent experiments (n = 4); and camphor (600 μM) was evaluated in three independent experiments (n = 3). Statistical analysis was performed using one-way ANOVA followed by Dunnett’s multiple-comparisons test. Differences were considered statistically significant at P < 0.05 (*), P < 0.01 (**), and P < 0.001 (***) versus the vehicle control.

## 3 Results

### 3.1 Molecular docking analysis of major rosemary monoterpenes at the orthosteric and allosteric sites of hSERT

Molecular docking simulations revealed substantial differences in the scoring functions and interaction profiles of fluoxetine and the major monoterpenes α-pinene, camphor, and 1,8-cineole within docking grids centered on the orthosteric S1 and allosteric S2 regions of the human serotonin transporter (hSERT) (Figure 1; Table 1). As expected, fluoxetine yielded the most favorable docking scores for hSERT among all compounds evaluated. At the orthosteric S1 site, fluoxetine exhibited a docking score of −11.2 kcal/mol, whereas docking within the S2 region yielded in a less favorable score of −8.2 kcal/mol. This represents a reduction in binding score between the two sites, indicating a more favorable predicted score within the S1 grid than within the S2 grid (Table 1). Structural analysis revealed that fluoxetine formed an extensive interaction network involving hydrogen bonds, salt bridges, π-cation interactions, and aromatic contacts with key residues lining the binding cavity, including Asp98, Tyr95, Ser438, Phe335, Lys490, and Glu494 (Figure 1A; Supplementary Figure S2; Table 1).

**FIGURE 1:**
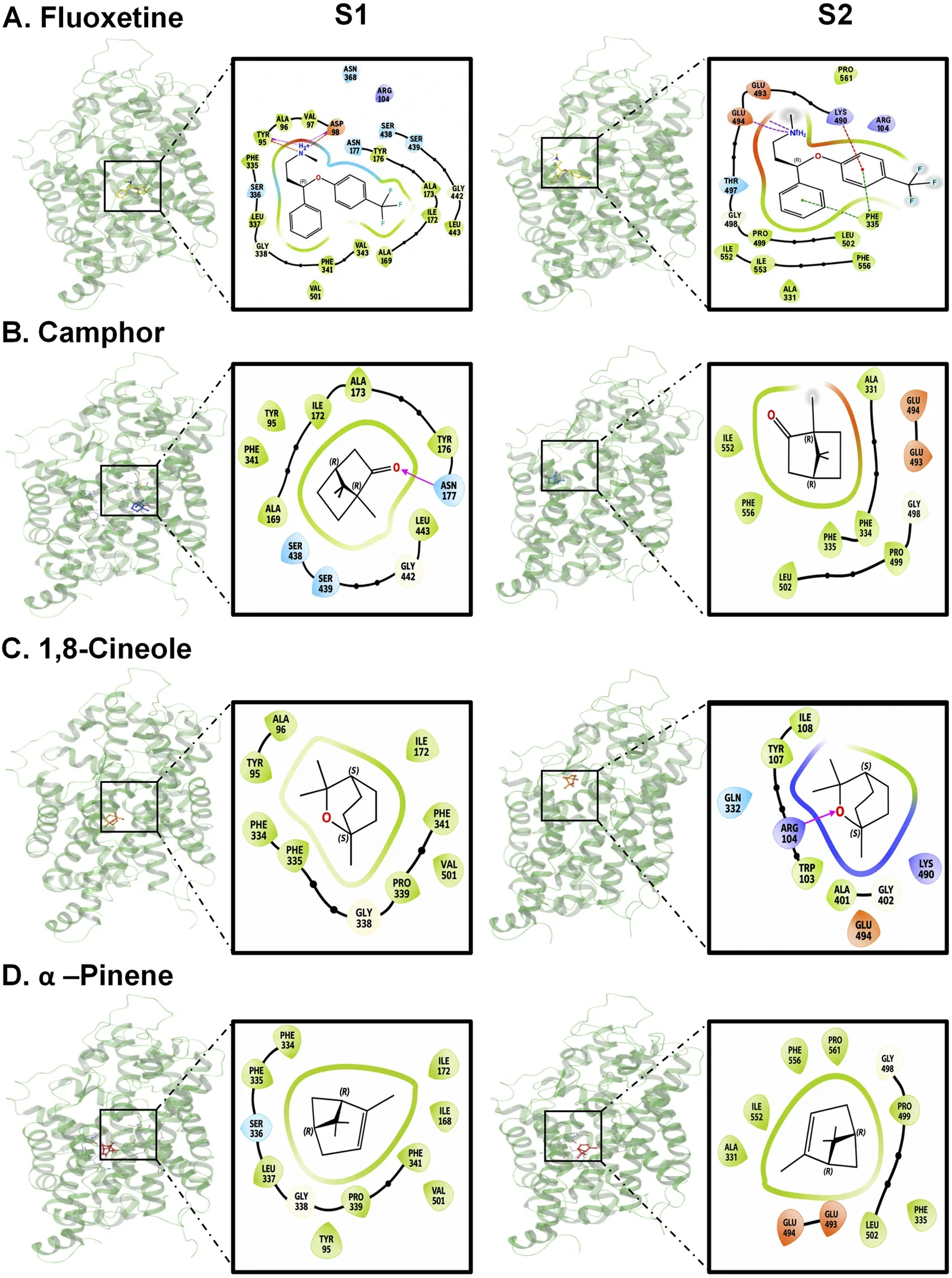
Predicted binding modes of camphor, 1,8-cineole, α-pinene, and fluoxetine at the S1 and S2 binding sites of human serotonin transporter (hSERT). Representative docking poses of the highest-ranked binding conformations obtained for fluoxetine **(A)**, camphor **(B)**, 1,8-cineole **(C)**, and α-pinene **(D)**. The left panels show the highest-ranked docking poses generated within the S1 grid of hSERT (PDB ID: 5I71), whereas the right panels show the corresponding predicted poses within the S2 grid (PDB ID: 5I73). Each panel includes a three-dimensional representation of the docked ligand within the transporter (left) and the corresponding two-dimensional interaction diagram (right), highlighting the amino acid residues predicted to contact the highest-ranked ligand poses. Docking simulations were performed using Glide XP in Schrödinger Maestro 14.3 after protein preparation with the Protein Preparation Wizard and ligand preparation with LigPrep/Epik (pH 7.0 ± 2.0) using the OPLS4 force field.

**TABLE 1:** Docking scores and predicted molecular interaction profiles of camphor, 1,8-cineole, α-pinene, and fluoxetine within docking grids centered on the S1 and S2 regions of the human serotonin transporter (hSERT).

Compound	Binding site	Docking score (kcal/mol)	Hydrogen bonds	Salt bridges	π-π Interactions	π-Cation Interactions	Aromatic H bond	Key interacting residues
Fluoxetine	S1	−11,2	2	1	-	1	1	Asp98, Tyr95, Ser438
Camphor	S1	−6,0	1	-	-	-	-	Asn177, Ile172, Tyr176, Phe341, Ser438, Leu443
1,8-Cineole	S1	−5,3	-	-	-	-	-	Tyr95, Ile172, Phe334, Phe335, Pro339, Phe341, Val501
α-Pinene	S1	−6.0	-	-	-	-	-	Tyr95, Phe334, Phe335, Pro339, Phe341, Val501
Fluoxetine	S2	−8.2	1	1	2	1	1	Phe335, Lys490, Glu494
Camphor	S2	−5.6	-	-	-	-	-	Ala331, Phe335, Glu494, Gly498, Pro499, Leu502, Ile552, Phe556
1,8-Cineole	S2	−3.1	1	-	-	-	-	Arg104, Ala169, Ile172, Ala173, Tyr176, Phe341, Ser438, Ser439, Gly442, Leu443
α-Pinene	S2	−5.0	-	-	-	-	-	Ala331, Phe335, Glu494, Gly498, Pro499, Leu502, Ile552, Phe556

Docking simulations were performed using Glide XP in Schrödinger Maestro 14.3 with the crystal structures of hSERT PDB ID: 5I71 (S1 site) and PDB ID: 5I73 (S2 site). Docking scores (kcal/mol), interaction types, and key interacting residues correspond to the highest-ranked predicted pose obtained for each ligand. Hydrogen bonds, salt bridges, π–π interactions, π–cation interactions, aromatic hydrogen bonds, and other ligand-residue contacts within the binding pocket are reported. A dash (−) indicates that no interaction of the specified type was identified.

In contrast, all three monoterpenes exhibited substantially weaker binding scores than fluoxetine at both binding sites. At the orthosteric S1 site, α-pinene and camphor showed identical docking scores (−6.0 kcal/mol), whereas 1,8-cineole displayed a slightly lower score (−5.3 kcal/mol), indicating lower predicted affinity for hSERT (Table 1). The highest-ranked camphor pose exhibited a distinct interaction pattern from the other monoterpenes. Although its docking score at the S1 site was identical to that of α-pinene (−6.0 kcal/mol), its carbonyl group enabled hydrogen-bond formation with Asn177 and Ser438 within the binding pocket (Figure 1B; Supplementary Figure S2; Table 1). In addition, the highest-ranked α-pinene pose was located within a hydrophobic region of the S1 grid and showed predicted hydrophobic and van der Waals contacts with by Tyr95, Ile172, Phe334, Phe335, Pro339, Phe341, and Val501 (Figure 1D; Supplementary Figure S2; Table 1). Similarly, highest-ranked 1,8-cineole pose showed predicted contacts predominantly with hydrophobic and aromatic residues within the S1 pocket (Figure 1C; Supplementary Figure S2; Table 1).

Differences among the monoterpenes were more evident at the allosteric S2 site. Camphor exhibited the most favorable docking score (−5.6 kcal/mol), followed by α-pinene (−5.0 kcal/mol) and 1,8-cineole (−3.1 kcal/mol). Compared with the S1 site, camphor showed only a slight reduction in docking score (−6.0 to −5.6 kcal/mol), whereas 1,8-cineole exhibited the largest decrease (−5.3 to −3.1 kcal/mol) (Table 1). Interaction analysis showed that the highest-ranked α-pinene and camphor poses were located in overlapping areas of the S2 grid and showed predicted contacts with hydrophobic residues, including Ala331, Phe335, Gly498, Pro499, Leu502, Ile552, and Phe556 (Figures 1B,D; Table 1). In contrast, 1,8-cineole was predicted to form a hydrogen bond with Arg104 at the S2 site (Figure 1C; Table 1).

Comparison of the docking scores yielded the following ranking of predicted binding favorability: fluoxetine ≫ camphor ≈ α-pinene > 1,8-cineole at both binding sites, consistent with the lower predicted affinity of the monoterpenes for hSERT (Table 1). These computational scores reflect the relative favorability of the predicted poses and should not be interpreted as experimental binding affinity values. Additionally, their interaction profiles are dominated by hydrophobic contacts and limited hydrogen-bond formation, indicating less favorable predicted interactions than those obtained for fluoxetine.

### 3.2 Major monoterpenes from *Rosmarinus officinalis* L. interact with hSERT and inhibit [^3^H]-paroxetine binding in a concentration-dependent manner

Interactions between the major monoterpenes of *R. officinalis* L. and hSERT were evaluated using [^3^H]-paroxetine radioligand displacement assays (Figure 2). α-Pinene, camphor, and 1,8-cineole inhibited [^3^H]-paroxetine binding in a concentration-dependent manner, producing sigmoidal concentration–response curves. Binding remained close to control levels at low concentrations (10^−10^–10^−7^ M) and progressively decreased at higher concentrations, reaching approximately 70%–80% inhibition at 10^−2^ M (Figure 2).

**FIGURE 2:**
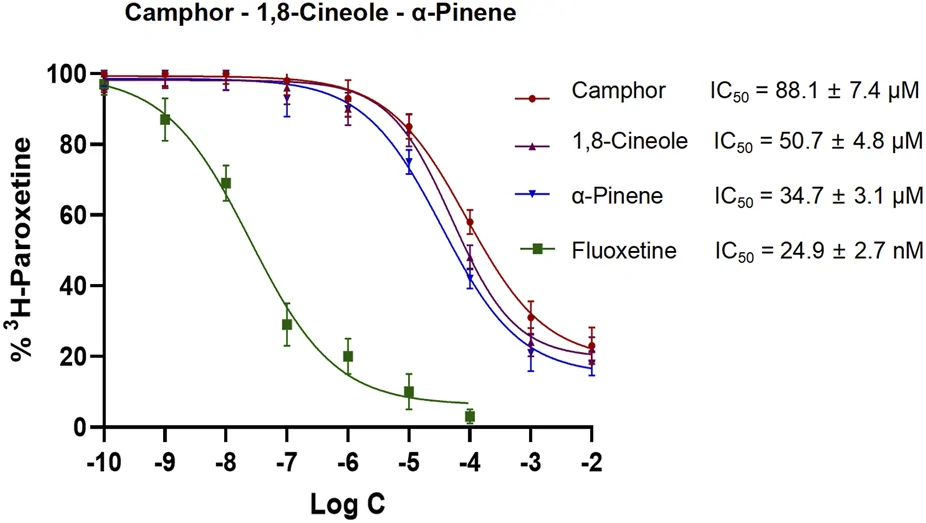
Concentration-dependent inhibition of [^3^H]-paroxetine binding to hSERT by the major monoterpenes of *Rosmarinus officinalis* L. HEK-SERT-YFP cell membranes were incubated with 2 nM [^3^H]-paroxetine in the presence of increasing concentrations (10^−10^–10^−2^ M) of camphor, 1,8-cineole, and α-pinene. Fluoxetine was included as a reference inhibitor. After 30 min of incubation at 27 °C, membrane-bound radioactivity was determined by rapid filtration followed by liquid scintillation counting. Binding is expressed as the percentage of specific [^3^H]-paroxetine binding relative to the vehicle control. Data are presented as mean ± SD from three independent experiments performed in triplicate. Concentration–response curves were fitted by nonlinear regression, and the inhibitory concentration (IC_50_) values are indicated in the graph.

Quantitative analysis showed that α-pinene was the most potent inhibitor of [^3^H]-paroxetine binding (IC_50_ = 34.7 ± 3.1 μM), followed by 1,8-cineole (50.7 ± 4.8 μM) and camphor (88.1 ± 7.4 μM). All IC_50_ values were within the low micromolar range, indicating substantially lower potency than classical SSRIs, which typically interact with hSERT in the nanomolar range (e.g., Fluoxetine IC_50_ of 24.9 ± 2.7 nM) (Figure 2). Although their displacement potency was substantially lower than that of fluoxetine, all three monoterpenes reduced [^3^H]-paroxetine binding under the assay conditions. The shallow concentration–response curves and incomplete displacement indicate a pharmacological profile distinct from fluoxetine, although the underlying mechanism remains unresolved.

### 3.3 Major monoterpenes from *Rosmarinus officinalis* L. partially inhibit hSERT-mediated substrate uptake

To determine whether the interaction of rosemary monoterpenes with hSERT is associated with changes in transporter activity, FFN246 uptake assays were performed in HEK293 cells expressing hSERT-YFP. Fluoxetine (0.5 μM) completely inhibited FFN246 uptake, whereas α-pinene, camphor, and 1,8-cineole produced partial inhibition (Figure 3A).

**FIGURE 3:**
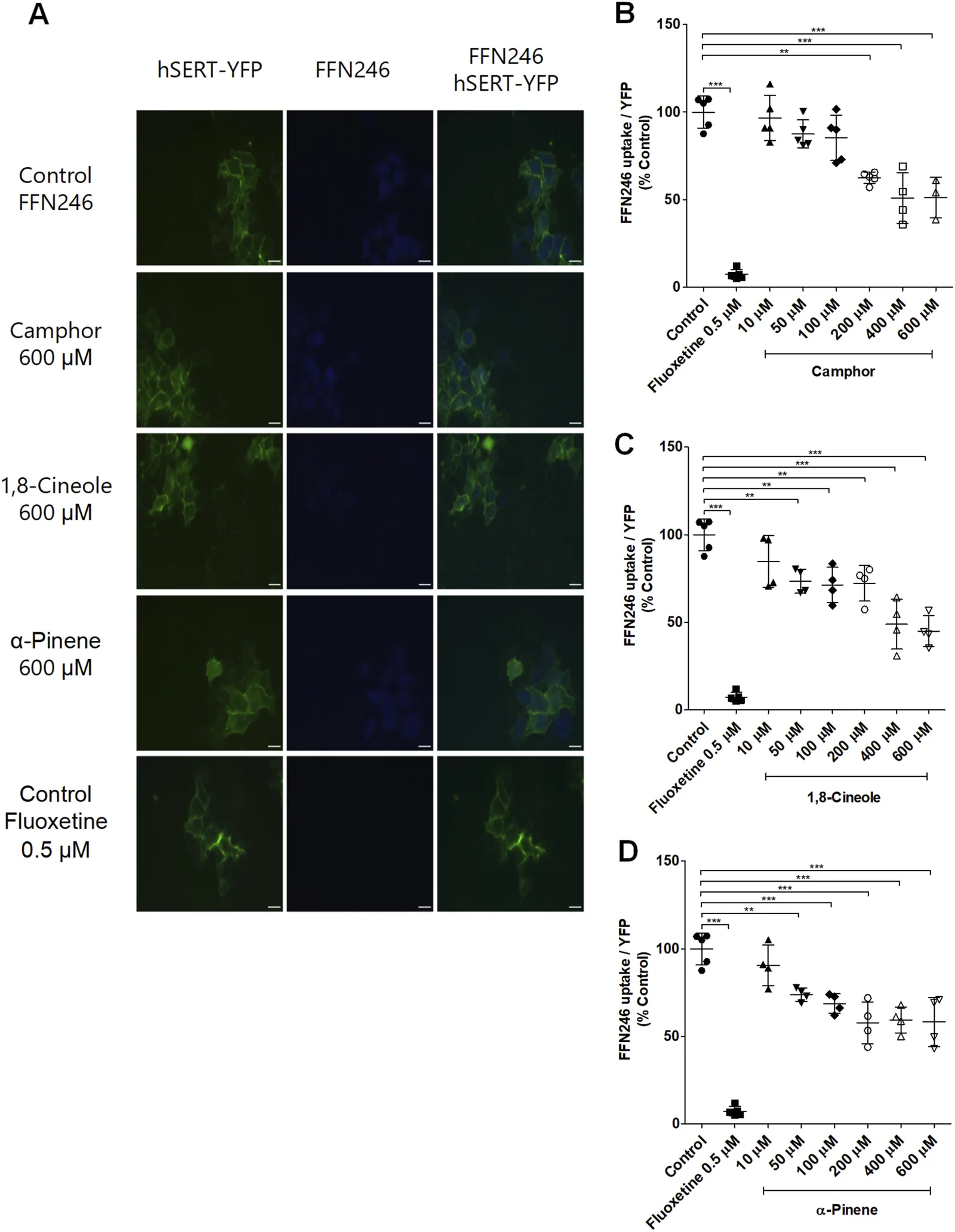
Functional inhibition of hSERT-mediated substrate uptake by major monoterpenes from *Rosmarinus officinalis* L. **(A)** Representative epifluorescence images of HEK-SERT-YFP cells showing SERT-YFP fluorescence (green), intracellular FFN246 fluorescence (blue), and merged images for vehicle-treated cells (control), cells treated with 600 μM camphor, 600 μM 1,8-cineole, 600 μM α-pinene, and 0.5 μM fluoxetine. Scale bar = 20 μm. Concentration-dependent effects of camphor **(B)**, 1,8-cineole **(C)**, and α-pinene **(D)** on hSERT activity in HEK-SERT-YFP cells. Cells were preincubated for 10 min with increasing concentrations (10–600 μM) of each monoterpene, followed by incubation with the fluorescent SERT substrate FFN246 (5 μM) for 20 min at 37 °C. Fluoxetine (0.5 μM) was used as a positive control of inhibition. Intracellular FFN246 fluorescence was normalized to SERT-YFP fluorescence (FFN246 uptake/YFP) and expressed as a percentage of the vehicle control. Data are presented as mean ± SD from at least three independent biological experiments. See Materials and Methods for sample sizes. Statistical analysis was performed using one-way ANOVA followed by Dunnett’s multiple-comparisons test. Statistical significance was set at P < 0.05 (*), P < 0.01 (**), and P < 0.001 (***) vs. vehicle control.

Quantitative analysis showed that α-pinene, camphor, and 1,8-cineole reduced FFN246 uptake in a concentration-dependent manner (Figures 3A–D). Unlike fluoxetine (0.5 μM; ∼93% inhibition), none of the monoterpenes completely inhibited transporter activity, reaching a maximum inhibition of 42%–55% at the highest concentration tested. FFN246 uptake progressively decreased as monoterpene concentrations increased from 10 to 600 μM. At 10 μM, inhibition was minimal (camphor ∼4%, α-pinene ∼10%, and 1,8-cineole ∼15%), whereas maximal inhibition (42%–55%) was observed at 600 μM (Figures 3A–D). Importantly, the observed inhibition did not exhibit the abrupt profile typically associated with competitive blockade by high-affinity ligands, such as fluoxetine, but rather a moderate concentration-response relationship consistent with partial inhibition under the experimental conditions.

Comparison of the functional uptake data with the radioligand binding experiments reveals an important pharmacological distinction. While all three monoterpenes effectively displaced [^3^H]-paroxetine in the low micromolar range, they maintain a nuanced influence on substrate transport, enabling partial inhibition even at high concentrations (up to 600 μM). This divergence may reflect a distinct mode of interaction with hSERT compared with the complete inhibition produced by conventional SSRIs; however, the involvement of specific transporter conformations remains hypothetical and requires direct experimental validation. Collectively, these results establish that the major monoterpenes present in *R. officinalis* L. essential oil interact with hSERT in radioligand-displacement assays and produce measurable effects on transporter-mediated substrate uptake. The partial, concentration-dependent inhibition observed for all three monoterpenes raises the possibility that these compounds may modestly modulate hSERT-mediated serotonin transport; however, the physiological relevance and underlying molecular mechanism remain to be established.

## 4 Discussion

The present study combines computational predictions with radioligand-displacement and functional uptake assays to investigate the interaction of the major monoterpenes from *R. officinalis* L. essential oil with hSERT. Molecular docking generated plausible poses for α-pinene, camphor, and 1,8-cineole within computational models of the S1 and S2 regions, with less favorable docking scores than fluoxetine. Radioligand-displacement and uptake assays independently provided experimental evidence of interaction with hSERT and partial inhibition of transporter function. However, these experiments did not determine whether the monoterpenes occupy either the S1 or S2 site under experimental conditions. Therefore, the docking results should be interpreted as site-specific structural hypotheses requiring direct experimental validation.

The less favorable docking scores obtained for the monoterpenes relative to fluoxetine are consistent with the established structural requirements for high-affinity hSERT inhibition (Masand and Gupta, 1999; Kristensen et al., 2011; Coleman et al., 2016; Coleman and Gouaux, 2018). Classical SSRIs possess protonatable amine groups and aromatic moieties that establish extensive electrostatic, hydrogen-bonding, and π-stacking interactions within the central binding cavity (Torres et al., 2003; Kristensen et al., 2011; Coleman et al., 2016; Coleman and Gouaux, 2018). In contrast, α-pinene, camphor, and 1,8-cineole are small, highly lipophilic molecules lacking ionizable groups and extended aromatic systems. Consequently, their predicted interaction profiles were dominated primarily by hydrophobic contacts and, in the case of camphor and 1,8-cineole, limited hydrogen-bond formation. The predominance of hydrophobic interactions likely explains both the relatively weak docking scores and the micromolar potency observed experimentally. Similar *in silico* less favorable interactions have been described for several plant-derived terpenoids acting on monoaminergic targets, supporting the notion that naturally occurring volatile compounds can interact with neurotransmitter systems through mechanisms distinct from those of conventional antidepressants (Fonseca et al., 2023; Guzmán-Gutiérrez et al., 2015; López et al., 2017; Weston-Green et al., 2021).

An interesting finding emerging from this study is the partial discordance between molecular docking predictions and experimental pharmacology. Although α-pinene and camphor yielded similar docking scores within the S1 grid, α-pinene exhibited substantially greater potency in radioligand binding assays and produced stronger inhibition of transporter-mediated substrate uptake. This discrepancy highlights an inherent limitation of static docking approaches, which primarily estimate thermodynamic binding favorability but do not fully account for protein conformational dynamics, ligand residence time, binding kinetics, or membrane-dependent effects (Gioia et al., 2017). The serotonin transporter undergoes complex conformational transitions during substrate translocation, and increasing evidence suggests that ligand efficacy may depend not only on binding affinity but also on the ability to stabilize specific transporter conformations (Kristensen et al., 2011; Zhong et al., 2012; Coleman et al., 2016; Coleman and Gouaux, 2018; Gradisch et al., 2024). Whether α-pinene differentially influences the conformational equilibrium of hSERT remains unknown and will require direct conformational and kinetic studies.

Another notable observation is that all monoterpenes displaced [^3^H]-paroxetine binding more efficiently than they inhibited FFN246 uptake. Whereas radioligand binding was markedly reduced at low micromolar concentrations, transporter-mediated substrate uptake remained only partially inhibited even at concentrations as high as 600 μM. This divergence indicates that the physical interaction of major monoterpenes from *R. officinalis* L. with hSERT does not necessarily correspond to complete functional inhibition. Instead, the data support a model in which rosemary monoterpenes act as partial functional inhibitors rather than classical competitive inhibitors. Several non-mutually exclusive mechanisms could explain the divergence between radioligand displacement and partial uptake inhibition, including incomplete transporter occupancy, kinetic effects, membrane partitioning, nonspecific membrane effects, or ligand-induced conformational changes (Gradisch et al., 2024; Niello et al., 2020; Zhong et al., 2012). Specifically, this discrepancy can be understood through the structural topology of hSERT: while paroxetine occupies the central orthosteric site (S1) to directly occlude the permeation pathway, the transporter also harbors a peripheral allosteric site (S2) in the extracellular vestibule, whose occupancy impedes ligand dissociation from S1 without necessarily arresting the full transport cycle (Coleman et al., 2016; Salomon et al., 2023), indicating that radioligand displacement alone does not necessarily imply engagement with a site obligatorily coupled to transport inhibition. A relevant precedent is ibogaine, which competes for SSRI binding while non-competitively inhibiting transport by stabilizing an inward-facing conformational state rather than sterically blocking the substrate pocket (Bulling et al., 2012; Coleman et al., 2019). Beyond direct site occupancy, the lipophilicity of these monoterpenes suggests a complementary, membrane-mediated mechanism, as small lipophilic molecules can accumulate in the lipid bilayer and perturb monoamine transporters, shifting the equilibrium between outward- and inward-facing conformations and modulating binding affinity and transport activity in non-proportional ways (Rudin et al., 2026; Uesono, 2026). In addition, the docking poses obtained within the S2 region raise the possibility of an allosteric interaction; however, docking alone cannot establish binding-site occupancy or functional allosteric regulation, and neither the radioligand displacement nor the uptake experiments performed here discriminate between orthosteric and allosteric mechanisms. Accordingly, the predicted S2 poses should be considered testable structural hypotheses rather than evidence of allosteric modulation. Confirmation will require site-directed mutagenesis within S2 residues, ligand-dissociation kinetic experiments, or other site-resolved pharmacological approaches (Plenge et al., 2021; Salomon et al., 2023).

The observation that 1,8-cineole and α-pinene exhibited the strongest functional effects despite their relatively modest predicted affinities is particularly intriguing. Previous studies have demonstrated anxiolytic- and antidepressant-like effects of essential oils rich in these monoterpenes in animal models (Ceremuga et al., 2017; Fonseca et al., 2023; Guzmán-Gutiérrez et al., 2015; Rahbardar and Hosseinzadeh, 2020). Rather than acting through a single pathway, the effects of these oils have often been attributed to a multi-target modulation involving GABAergic, dopaminergic, and glutamatergic signaling (Fonseca et al., 2023; López et al., 2017; Rahbardar and Hosseinzadeh, 2020; Weston-Green et al., 2021). The present findings expand this mechanistic framework by suggesting that the interaction of rosemary monoterpenes with hSERT may contribute to the reported behavioral effects, although their physiological relevance remains to be established. The present findings extend this framework by identifying hSERT as a potential molecular target of these monoterpenes *in vitro*. However, because behavioral, neurochemical, and *in vivo* outcomes were not assessed, the present data do not establish a causal relationship between hSERT inhibition and the reported antidepressant-like effects of rosemary. Instead, the interaction of rosemary monoterpenes with hSERT should be considered one possible component of a broader, likely multi-target neuropharmacological profile that requires direct validation in appropriate *in vivo* models.

To our knowledge, this study represents the first comprehensive characterization integrating molecular docking, radioligand binding, and functional uptake assays to investigate the interaction between major rosemary monoterpenes and hSERT. Previous reports have largely focused on behavioral outcomes or indirect neurochemical effects of rosemary extracts and essential oils, without directly addressing transporter pharmacology (Fonseca et al., 2023; Machado et al., 2009; 2013; Niu et al., 2025; Rahbardar and Hosseinzadeh, 2020). By providing experimental evidence that α-pinene, camphor, and 1,8-cineole interact with hSERT and partially inhibit transporter function, the present work expands current understanding of the molecular mechanisms underlying the neuropsychopharmacological properties of *R. officinalis* L. and identifies hSERT as a previously underappreciated molecular target of rosemary-derived monoterpenes.

Several limitations of the present study should nevertheless be acknowledged. First, the docking analyses relied on static crystallographic structures of hSERT and therefore do not fully capture the conformational flexibility inherent to transporter function (Coleman et al., 2016; Gioia et al., 2017). Molecular dynamics simulations could provide additional insights into ligand stability, residence time, and conformational effects (Gioia et al., 2017); whereas site-directed mutagenesis of residues lining the S1 and S2 regions could test the proposed site-specific interactions. Second, all functional experiments were performed in heterologous HEK293 cells overexpressing hSERT, a reductionist system that may not fully reproduce the regulatory complexity of serotonergic neurons *in vivo* (Kristensen et al., 2011; Torres et al., 2003). Available pharmacokinetic evidence supports central nervous system penetration of these lipophilic monoterpenes but does not show that concentrations approaching 600 μM are attained in the human brain. After a single 300-mg oral dose of a standardized monoterpene preparation, peak human plasma 1,8-cineole concentrations were 167.6–518.9 ng/mL (approximately 1.1–3.4 μM) (Zhao et al., 2016). In mice inhaling an α-pinene-rich essential oil for 90 min, total regional brain α-pinene concentrations reached 3.0–18.7 μg/g (approximately 22–137 nmol/g tissue) (Kasuya et al., 2015), whereas orally administered 1,8-cineole was rapidly detected in rat brain (Dao et al., 2023); comparable quantitative data for human brain and camphor are lacking. Thus, although the lower experimental range may overlap preclinical total-tissue exposure, 600 μM should be considered an *in vitro* upper bound for defining maximal hSERT inhibition rather than an established physiologically attainable exposure. Direct brain pharmacokinetic/pharmacodynamic studies are required to determine the contribution of this mechanism to rosemary’s neurobehavioral effects. Finally, given the pleiotropic pharmacological profile of monoterpenes, additional studies are required to determine the relative contribution of hSERT modulation to the behavioral effects attributed to rosemary preparations, which are likely mediated through multiple neurotransmitter systems and signaling pathways (Fonseca et al., 2023; López et al., 2017; Rahbardar and Hosseinzadeh, 2020).

Despite these limitations, the integration of computational, binding, and functional approaches provides robust evidence that the major monoterpenes of *R. officinalis* L. act as partial inhibitors of hSERT. These findings reveal a previously unrecognized mechanism through which rosemary-derived compounds influence serotonergic neurotransmission and provide a mechanistic basis for future studies exploring their therapeutic potential in neuropsychiatric disorders.

## Data Availability

The raw data supporting the conclusions of this article will be made available by the authors, without undue reservation.

## Supplementary material

SUPPLEMENTARY FIGURE S1 Validation of the molecular docking protocol by redocking of citalopram into the orthosteric (S1) and allosteric (S2) binding sites of hSERT. **(A)** Superposition of the crystallographic citalopram (magenta) and the highest-ranked redocked pose (dark gray) in the S1 binding site of hSERT (PDB ID: 5I71), yielding an RMSD of 0.91 Å. The lower panel shows the overlay of both poses within the S1 binding pocket, highlighting representative interacting residues. **(B)** Superposition of the crystallographic citalopram (purple) and the highest-ranked redocked pose (dark gray) in the S2 allosteric binding site of hSERT (PDB ID: 5I73), yielding an RMSD of 0.67 Å. The lower panel shows the overlay of both poses within the S2 binding pocket, highlighting representative interacting residues.

SUPPLEMENTARY FIGURE S2 3D-Predicted binding poses of the major monoterpenes of Rosmarinus officinalis L within the S1 and S2 binding sites of hSERT. **(A–D)** show the highest-ranked docking poses of camphor, 1,8-cineole, α-pinene, and fluoxetine, respectively, in the orthosteric S1 binding site of hSERT (PDB ID: 5I71). **(E–H)** show the corresponding poses in the allosteric S2 binding site (PDB ID: 5I73). Ligands are displayed as sticks within the protein binding pocket, and green dashed lines indicate the predicted intermolecular interactions between the ligands and surrounding residues.

SUPPLEMENTARY FIGURE S3 Camphor, α-pinene, and 1,8-cineole show no significant effect on HEK293 cells viability. Cells were exposed to increasing concentrations (50 – 600 µM) of each monoterpene for 30 min, and cell viability was assessed using the MTT assay. Data are presented as mean ± SD from three independent biological experiments. Statistical analysis was performed using one-way ANOVA followed by Dunnett’s multiple-comparisons test. No statistically significant differences were observed compared with the vehicle control.
